# Hierarchical habitat-use by an endangered steppe bird in fragmented landscapes is associated with large connected patches and high food availability

**DOI:** 10.1038/s41598-019-55467-2

**Published:** 2019-12-12

**Authors:** Julia Gómez-Catasús, Vicente Garza, Manuel B. Morales, Juan Traba

**Affiliations:** 1 Terrestrial Ecology Group, Department of Ecology, Universidad Autónoma de Madrid (TEG-UAM), C/Darwin 2, 28049 Madrid, Spain; 2 Centro de Investigación en Biodiversidad y Cambio Global, Universidad Autónoma de Madrid (CIBC-UAM), C/Darwin 2, 28049 Madrid, Spain; 3 C/Vía Límite 29, 28029 Madrid, Spain

**Keywords:** Conservation biology, Grassland ecology

## Abstract

Multidimensional approaches must be employed when addressing habitat use patterns. In this study, we aim to elucidate the hierarchical nature of space use by species inhabiting fragmented landscapes, using the threatened Dupont’s lark (*Chersophilus duponti*). The intensity of space use by Dupont’s lark was estimated using the Kernel Density Function on territory locations in 2015. We measured descriptors of habitat quality at metapopulation (connectivity and patch size), landscape (land-use types and anthropogenic disturbance) and microhabitat-scale (plant structure and composition, herbivore abundance and food availability) at 37 sampling stations. We fitted a Partial Least Squares Regression (PLSR) which yielded two components, accounting for 81% of total variance. Metapopulation-scale factors had the greatest explanatory power (32%), followed by microhabitat (17%) landscape (10%) and spatial predictors (3.6%). Connectivity and patch size were key factors explaining habitat use, and wind farms had a negative effect. At microhabitat-scale, space use was positively associated with *Coleoptera, Orthoptera, Araneae* and *Diptera* biomass, but negatively with *Formicidae* and *Blattodea* biomass, the cover of *Stipa spp, Koeleria vallesiana* and moss. This research highlights the hierarchical nature of habitat use in fragmented landscapes. Therefore, conservation measures should ensure connectivity, guarantee a minimum patch size, and improve habitat quality within patches.

## Introduction

Habitat selection processes have a hierarchical nature^1^ and the factors governing habitat selection processes and habitat-use patterns occur consequently across different spatial scales. At microhabitat scale, main factors are related with individual requirements (i.e. food availability, vegetation structure)^2^, whereas landscape structure and factors determining population dynamics are crucial at larger spatial scales (e.g. isolation, connectivity)^3^. Therefore, studies addressing hierarchical processes must take into account environmental factors working at the different spatial scales.

Metapopulation theory is broadly employed in the analysis of factors affecting spatial and temporal dynamics of fragmented populations^4^. Classic metapopulation models assume that patch size and connectivity are two crucial environmental factors to explain occupancy processes, where small and isolated patches will have a lower probability of occupancy than large and connected ones^4,5^. A different approach assumes that habitat patch quality is the most critical factor influencing habitat-use patterns, since it impacts on population viability^6^. Originally, metapopulation theory and habitat quality studies were two non-integrated paradigms in conservation biology. More recently, parameters of habitat quality measured at smaller spatial scales have been integrated in metapopulation models, conditioning the effects of classic metapopulation factors^6–8^.

In this study, we employed a multiscale approach to disentangle the hierarchical nature of habitat use by species inhabiting fragmented landscapes. We focused on steppe habitats, which have been subjected to an accelerated process of fragmentation during the last decades^9,10^, leading to a metapopulation scenario for species strictly depending on these habitats. Specifically, we used the threatened Dupont’s lark *Chersophilus duponti* (Vieillot 1820), a habitat specialist occupying flat shrub-steppes^11^ and with insectivorous habits^12^, as a model species. In fragmented habitats, the distribution of Dupont’s lark is influenced by the size of the patches, their degree of connectivity and the landscape matrix^8^. At microhabitat scale, vegetation structure is a key descriptor of habitat quality, inhabiting short shrubs and avoiding dry pastures and crops^11,13^. However, the absence of Dupont’s lark in areas meeting these requirements and with apparently optimal habitat for the species suggests that other critical resources might play an important role on the definition of habitat patch quality, determining the observed habitat-use patterns.

We integrated several descriptors of habitat quality measured at three spatial scales: metapopulation, landscape and microhabitat. At metapopulation scale, we incorporated connectivity and patch size in accordance with the metapopulation theory. At landscape scale, we considered land uses and two sources of anthropogenic pressure: (1) wind farms since they have deleterious impacts on Dupont’s lark populations^14^; and (2) crops due to their fragmentation effect on shrub-steppes^15,16^. At microhabitat scale, we considered vegetation structure and floristic composition, two key factors determining Dupont’s lark distribution^11^. In addition, we included herbivore abundance due to the effects of both wild and domestic herbivores on plant community structure and diversity in steppe-ecosystems^17^. Changes in herbivory pressure and specifically, the abandonment of livestock grazing, favours the encroachment of higher shrubs^16^ and might reduce food availability (e.g. dung beetles)^18^, potentially impacting on the habitat-use pattern by Dupont’s lark^16^. Lastly, we incorporated food availability in terms of arthropod biomass, including epigean, flying and dung-dwelling arthropods. Epigean and flying arthropods have been described as important groups in the diet of Dupont’s lark^12,19^, and coprophagous arthropods seem to be crucial in Dupont’s lark populations with high livestock density^20^.

The goal of this study is to evaluate the relative importance of the above-mentioned factors acting at different spatial scales, on habitat-use patterns of Dupont’s lark in a highly fragmented landscape. In accordance with a previous study^8^, we expect that space use will be favoured by high connectivity and habitat availability, whereas it will be negatively affected by human disturbance and the presence of land-use types not suitable for the species (i.e. afforestations, ploughings or crops). Further, we predict that areas with greater herbivore abundance, higher food availability and with optimal plant structure and floristic composition (i.e. high pillow-shaped, low shrub cover such as *Genista sp*. and *Thymus sp*.)^11^ will be intensively used by Dupont’s lark. The obtained results may contribute to improve the knowledge of the mechanisms determining the distribution pattern of Dupont’s lark, indispensable to establish conservation measures that curb the overall decline undergone by this endangered species^21^. In synthesis, we aim to expand the understanding of factors driving habitat use patterns in species inhabiting fragmented landscapes and provide a useful approach to address future studies focused on hierarchical processes or patterns in ecology.

## Material and Methods

The ethics committee of Animal Experimentation of the Autonomous University of Madrid as an institution enabled by the Community of Madrid (Resolution 24th September 2013) for the evaluation of projects based on the provisions of Royal Decree 53/2013, 1st February, has provided full approval for this research (CEI 80-1468-A229). All experiments were performed in accordance with relevant guidelines and regulations.

### Study area

**Figure 1:**
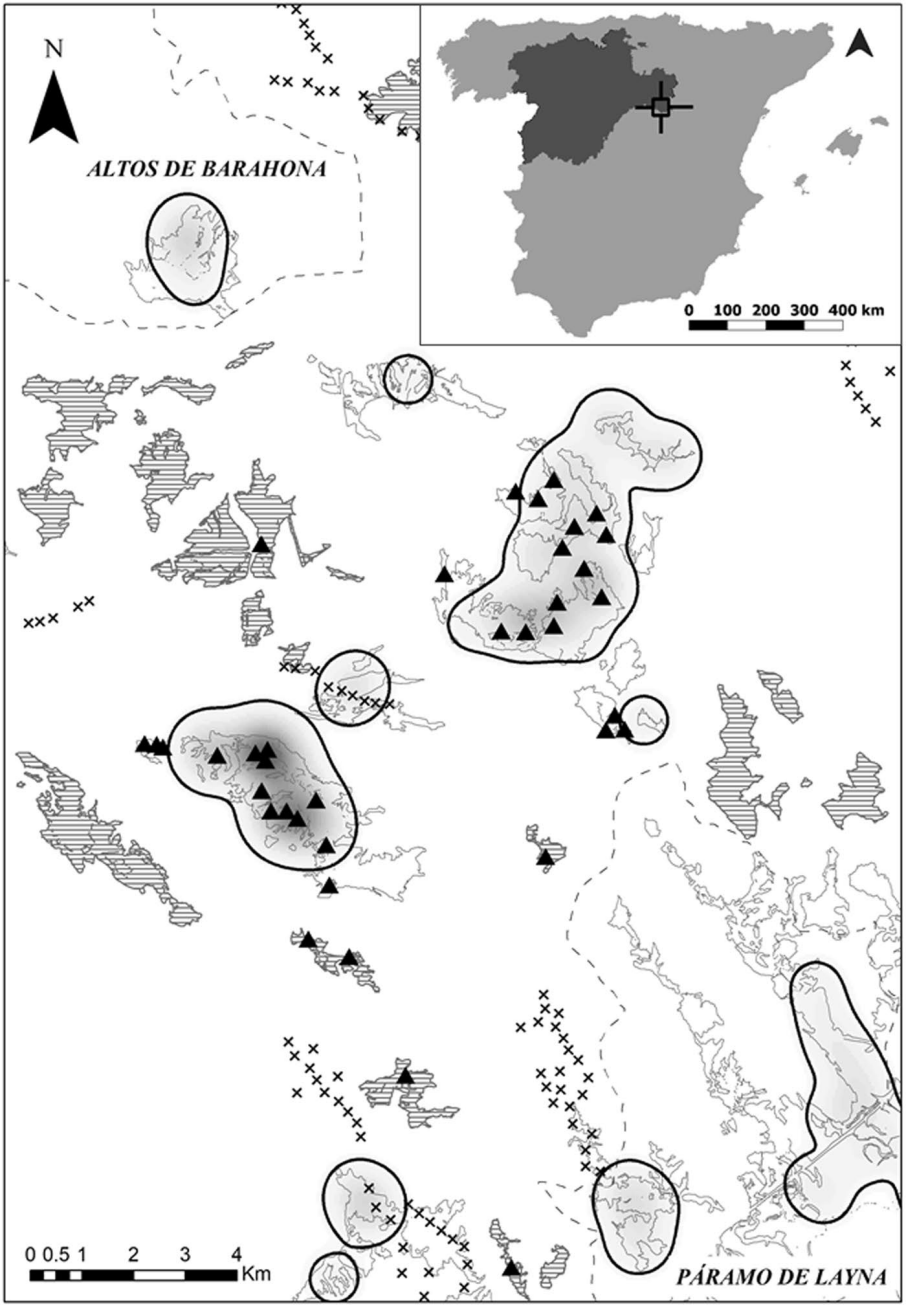
Study area “La Tierra de Medinaceli” region (Soria, central Spain). The map illustrates a gradient in the intensity of space use by Dupont’s lark estimated by the Kernel Density Function: from high (dark grey) to low intensity of space use values or absences (white). The patches of optimal habitat with presence (grey lines) and absence (stripes) of Dupont’s lark, the 95% volume contour (i.e. 95% of the volume of the probability density function; black lines), the wind turbine locations (crosses) and the sampling stations (triangles), are depicted. The name of the SPAs (capital letters) and their limits (dashed line), are shown.

The study area is “La Tierra de Medinaceli” region, covering 1126 km^2^ (02° 26′ 35.1″O; 41° 11′ 28.9″N). It is located in the Soria province (central Spain), and constitutes an independent Dupont’s lark subpopulation^22^. The “Páramo de Layna” Special Protecting Area (SPA) is part of this subpopulation, whereas most of the surface of the “Altos de Barahona” SPA belongs to another subpopulation^22^ (except for the most eastern locality; Fig. 1). The study area is characterised by a plateau landscape (1150 metres above sea level), covered by short shrubs such as *Genista pumila, G. scorpius, Thymus spp*. and *Linum suffruticosum*^11^. Crops, ploughed fields and pine afforestations are interspersed in the area. The climate is Continental Mediterranean, with a mean temperature of 10.6 °C and a mean annual rainfall of 500 mm.

The study area is comprised of 25 localities with optimal habitat for Dupont’s lark (i.e. flat shrub-steppes with predominance of *G. pumila* and slope values between 12 and 15%)^11^. The total surface of optimal habitat per locality varies from 0.78 up to 2648.24 ha (mean ± SD = 201.37 ± 518.00 ha). Localities are fragmented due to natural (geological) causes and/or anthropogenic pressures (Fig. 1), but patches of optimal habitat closer than 1 km are considered the same locality since most adult movements occur within this distance^23^. Patch size varies between 0.09 and 1028.09 ha (mean ± SD = 18.65 ± 75.68 ha). Dupont’s lark occurred during 2015 in 14 out of the 25 localities, whereas local extinction events took place recently in 4 localities and the occurrence of the species was not recorded during the last 8 years in the remaining 7 localities (own unpubl. data).

### Dupont’s lark surveys

We carried out Dupont’s lark surveys by foot transects during the breeding season (from the end of March until the middle of June) from 2008 to 2015 (detailed methodology can be found in Suárez^24^). The number of transects per patch was proportional to patch size (ranging between 1 and 19 transects per patch) and their length varied between 1–3 kilometres. Each transect was walked at least 4 times per breeding season, alternating the starting point in each visit. Surveys were carried out approximately 1 hour before dawn (from ca 5:00 h in March to ca 3:00 h in June, solar hour) and they lasted around 1 hour. The locations of singing males were georeferenced with a GPS and the territory mapping method^25^ was employed to locate Dupont’s lark territories. Territories were defined each year gathering observations from different visits and differentiating simultaneously contacted neighbouring males. This census methodology has been broadly employed and tested as the most adequate technique for monitoring Dupont’s lark populations^26^.

### Location of habitat sampling stations

We located 37 sampling stations covering the whole gradient in intensity of space use by Dupont’s lark from 2008 to 2014 (Fig. 1; see ‘Statistical analyses’). Sampling stations were placed attending to three factors: (1) the absence of wind farms within the patch due to the negative effect of these infrastructures on Dupont’s lark populations^14^; (2) the presence of optimal habitat for the species (i.e. short shrub with slopes lower than 15%)^11^; and (3) keeping 400 m of minimum distance between sampling stations in accordance with the maximum home range recorded for the species (37 ha)^11^.

### Variables at metapopulation scale

We used a relative connectivity index (RC), estimated as the distance from each sampling station to the centroid of Dupont’s lark territories in the nearest population (Table 1). All Dupont’s lark territories separated by less than 1 km were considered to belong to the same population^8^. Lastly, we estimated patch size as the surface of continuous optimal habitat for the species (i.e. shrub-steppe with slope lower than 15%) where the sampling stations were located. These variables were calculated with the software QGIS 2.14.0^27^.

**Table 1:** Response variable analysed and habitat quality measures at metapopulation, landscape and microhabitat scale incorporated in the analysis. Moreover, the generated spatial predictors based on a third-degree polynomial of the geographic coordinates (spatial), are shown. Applied transformations are specified.

Scale	Environmental Factor	Variables	Transformation
Response	Intensity of space use by Dupont’s lark in 2015	Kernel Density Function (KDF)	$\sqrt{x}$
Metapopulation	Connectivity	Relative connectivity RC (distance to the nearest occupied population) (km)	None
	Patch size	Surface of optimal habitat (i.e. shrub-steppe with slope lower than 15%) (ha)	*log*(*x*)
Landscape	Proximity to sources of anthropogenic pressure	Distance to the nearest crop (km)	*log*(*x*)
		Distance to the nearest wind turbine (km)	None
	Land-use types	Shrub-steppe with slope <15% (%), Shrub-steppe with slope > 15% (%), Pastures (%), Crops (%), Ploughings (%), Afforestations (%), Infrastructures (%)	$arcos\,(\frac{x+1}{100})$
Microhabitat	Food availability (biomass)	*Coleoptera* (mg), *Blattodea* (mg) and *Hymenoptera* family *Formicidae* (mg)	*log*(*x* + 1)
		*Orthoptera* (mg) and Coprophagous arhtropods (mg)	$\sqrt{x}$
		*Diptera* (mg) and *Araneae* (mg)	None
	Herbivore abundance	Dung counts	$\sqrt{x}$
	Horizontal plant structure	Total vegetation cover (%) and Shrub cover (%)	$arcos\,(\frac{x+1}{100})$
		Bare ground cover (%), Rock cover (%), Lichen cover (%), Moss cover (%), Detritus cover (%), Perennial herbaceous cover (%) and Annual herbaceous cover (%)	*ln*(*x*)
	Vertical plant structure	Maximum modal height (cm) and Number of contacts at 0–5 cm, 5–10 cm, 10–30 cm and above 30 cm height	$\sqrt{x}$
	Floristic composition	Individual cover of perennial species (both woody and herbaceous) (%)	None
Spatial	Geographic coordinates	Third-degree polynomial: X, Y, XY, X^2^, Y^2^, X^2^Y, XY^2^, X^3^, Y^3^	None

### Variables at landscape scale

At the landscape scale, we incorporated the proximity to sources of anthropogenic pressure (wind farms and crops) and the availability of different land-use types (Table 1). First, we measured the distance from each sampling station to the nearest wind turbine and the nearest crop. Secondly, we measured the percentage of surface of each land-use type in a 150 m buffer around each sampling station, based on the mean home range of Dupont’s lark (9 ha)^11^. All these variables were calculated with the software QGIS 2.14.0^27^.

### Variables at microhabitat scale

At microhabitat scale, we measured food availability, vegetation structure, floristic composition and herbivore abundance (see scheme of sampling stations in Supplementary Fig. S1; Table 1).

#### Food availability

We measured epigean, flying and coprophagous arthropod biomass at the beginning (April), mid (May) and end (June) of the breeding season in 2015 (Table 1). Epigean arthropod community was sampled in three pitfall traps per sampling station placed at 5 meters intervals. Each trap consisted of a transparent plastic cup of 230 ml, 7 cm diameter and 10 cm depth, with several holes at the top easing drainage in case of rain. Plastic cups were buried and protected by a PVC tube to prevent its collapse. Each trap was filled with 175 ml of 40% ethylene glycol and a drop of soap to reduce surface tension^28^. Traps were placed in the absence of precipitations and with a mean temperature of 8.7 °C, 12.5 °C and 17.2 °C in April, May and June sampling, respectively. After a week the traps were filtered and the individuals collected were stored in 70% ethanol. This method has been tested in several studies being an efficient technique for capturing a wide spectrum of epigean arthropods (see for example Traba *et al*.^29^). Secondly, flying arthropods were sampled in two 10 m transects per sampling station employing an entomological sweep net. The individuals collected were stored in the same bottle as epigean arthropods with 70% ethanol. Lastly, coprophagous arthropods were sampled once at the end of the breeding season (June). At each sampling station, we placed one pitfall trap for coprophagous insects, which consisted of a 20 cm diameter plastic container, baited with 200 g of dung from local livestock. Traps were active during one day under similar weather conditions in all sampling stations. Coprophagous arthropods were stored in 70% ethanol and only those individuals with coprophagous habits were identified: order *Coleoptera* family *Scarabeidae* (*Gymnopleurus sp., Onthophagus sp*. and *Scarabeus sp*.) and order *Diptera* suborder *Brachycera*.

Arthropods were determined to its taxonomic order. Body length was measured (excluding legs, antennas and other appendices) to estimate arthropod biomass employing 53 specific equations from Hódar^30^:

1 $$W=\alpha \cdot B{L}^{b}$$

where *W* is arthropod biomass in mg, *BL* is body length and *a* and *b* are specific parameters for each group (or each order)^30^. When possible, body length was measured in a maximum of 15 individuals per sample. To minimize observer bias, all samples were identified by the same researcher (JGC).

In each sampling period, biomass per order was estimated as the mean biomass in the active pitfall traps (usually three per sampling station). Subsequently, total biomass per order was estimated as the sum of the means of the three sampling periods (April, May and June). Lastly, coprophagous arthropod biomass was estimated as the biomass measured in the corresponding sampling in June.

#### Herbivore abundance

We counted herbivore droppings in three quadrats of 2 × 2 m per sampling station (Table 1). Droppings were identified at the species level (European rabbit, Iberian hare, roe deer and sheep) and herbivore abundance was estimated as the average dung counts in the three quadrats per sampling station. This value was used as a proxy of herbivore abundance.

#### Vegetation structure and floristic composition

We carried out only one vegetation sampling in June when the whole plant community was identifiable, both woody and herbaceous species. Vegetation sampling was carried out in three quadrats of 1 × 1 metres per sampling station. Regarding the vertical structure we measured: (i) maximum modal height; and mean number of contacts in five sampling points per quadrat at (ii) 0–5 cm; (iii) 5–10 cm; (iv) 10–30 cm; and (v) above 30 cm height (Table 1). Regarding the horizontal structure we measured: (i) total vegetation cover (%); (ii) bare ground cover; (iii) rock cover; (iv) woody plant cover; (v) perennial herbaceous cover; (vi) annual herbaceous cover; (vii) detritus cover; (viii) moss cover: and (ix) lichen cover (Table 1). Lastly, in order to estimate floristic composition, we recorded horizontal cover in the 1 × 1 m quadrat of all perennial species (both woody and herbaceous; Table 1). For each variable (structural or compositional), we used the mean value of the three quadrats per sampling station.

### Statistical analysis

The Kernel Density Function (KDF) was used to estimate the intensity of space use by Dupont’s lark. KDF is a two-dimensional representation of the relative frequency distribution of a spatial pattern of points^31^. The KDF assigns a higher probability value to those areas with a greater number of points (i.e. Dupont’s lark territories) and their fit to data points depends on the smoothing parameter^31^. We estimated the KDF using the Hawth’s Analysis Tools for ArcGIS 9.3^32^, with a smoothing factor of 500 and a cell size of 50 × 50 m, according to Dupont’s lark home range^11^. Employing these parameters, the 95% and 80% volume contours (i.e. 95% and 80% of the volume of the probability density function) are reached at 776.96 ± 185.41 m and 526.38 ± 218.05 m (mean ± SD) from Dupont’s lark territories (Fig. 1). This KDF configuration is in agreement with Dupont’s lark movement behaviour, since most within-territory movements occur within 1 km distance^23^.

We estimated two KDFs: (1) employing all territories accumulated during 2008–2014 to obtain an indicative value of the intensity of space use by Dupont’s lark, which was used as criterion to select locations of sampling stations (see ‘Location of habitat sampling stations’); and (2) employing only territories in 2015 to obtain the intensity of space use in 2015, which was the response variable in subsequent analyses. This variable was estimated as the mean value in a 150 m buffer around each sampling station, according to the mean home range of 9 ha described for the species^11^.

Variables were transformed when necessary to achieve linearity (Table 1) and all quadratic relationships were incorporated. Moreover, we incorporated only those arthropod orders with a mean contribution higher than 4% of the total biomass in order to incorporate those relevant groups in the diet of Dupont’s lark nestlings^19^: *Coleoptera* (30.2%), *Diptera* (20.5%), *Hymenoptera* – *Formicidae* (15.8%), *Orthoptera* (10.8%), *Blattodea* (5.3%) and *Araneae* (4.4%). Due to the high number of ants recorded, the order *Hymenoptera* was subdivided in *Formicidae* and non-*Formicidae*. The latter was not incorporated in the analysis (mean contribution to total biomass 1.4%).

We performed Principal Component Analyses (PCA) to obtain synthetic and independent environmental gradients that were incorporated as predictors in subsequent analyses. We carried out 4 PCAs, one on each of the following sets of variables: (i) land-use types; (ii) horizontal plant structure; (iii) vertical plant structure; and (iv) floristic composition. A covariance matrix was employed when the unit of measure was the same for all variables (i.e. land-use types, horizontal vegetation structure and floristic composition) and a correlation matrix when the units of measure differed (i.e. vertical vegetation structure). In the case of the floristic composition, we removed rare species recorded in less than 10% of the sample (i.e. 4 sampling stations). We retained those components explaining at least 50% of total accumulated variance. PCAs were fitted using IBM SPSS Statistics 21 software^33^.

We carried out Partial Least Squares Regression (PLSR) to analyse the effect of the explanatory variables measured at metapopulation, landscape and microhabitat scale on the intensity of space use by Dupont’s lark. Moreover, we generated spatial predictors based on a third-degree polynomial of the geographic coordinates that were incorporated in the PLSR model in order to control for potential spatial dependencies (Table 1)^34^. PLSR is an extension of multiple regression analysis, which tests for associations between the response variable and linear combinations of predictors that maximise the variance explained in the response variable^35^. It is highly robust to small sample sizes and high number of predictors (i.e. overfitting) and severe multicollinearity^35^. In this study, the low sample size (n = 37 sampling stations), high number of predictors (51) and the multicollinearity between predictors (see Supplementary Tables S1, S2), supports the use of this approach. The relative contribution of each predictor to the PLSR component was estimated by means of the square of predictor weights, which are significant when they are greater than the threshold value *1/number of predictors* (see for example Sánchez-Oliver *et al*.^36^ or Morales *et al*.^37^). PLSR was fitted using the package plsdepot^38^ of the free R software (v. 1.0.143)^39^ and only those components significant after a tenfold validation procedure were retained.

## Results

The PCA on land use variables yielded one component *PC1-Land* (58.67% of total variance), which defined a gradient of habitat availability for Dupont’s lark. It opposed shrub-steppe surface with slope lower than 15% (correlation coefficient: 0.978) to shrub-steppe surface with slope higher than 15% (−0.807). The PCA on horizontal vegetation structure variables yielded 2 components (60.37% of total variance), which explained 35.63% and 24.74% of total variance, respectively. The first component *PC1-Hor* defined a positive gradient of moss cover (0.920) and the second component *PC2-Hor* a positive gradient of rock cover (0.776). The PCA on the vertical vegetation structure variables yielded 2 components (71.68% of total variance) explaining 43.45% and 28.23% of total variance, respectively. The first component *PC1-Ver* was positively associated with the number of contacts at 5–10 cm height (0.843) and the number of contacts at 10–30 cm height (0.864). The second component *PC2-Ver* was positively related with the number of contacts above 30 cm height (0.783). Lastly, the PCA on the floristic composition variables yielded 4 components (56.22% of total variance) explaining 22.79%, 13.29%, 11.87% and 8.27% of total variance, respectively. The first component *PC1-Flor* opposed *Thymus vulgaris* (0.833) to *Thymus zygis* cover (−0.890). The second component *PC2-Flor* defined a negative gradient of *Thymus mastigophorus* cover (−0.817). The third component *PC3-Flor* was negatively associated with the *Stipa* genus cover (−0.843) and the fourth component *PC4-Flor* was positively related with *Koeleria vallesiana* cover (0.695) (see Supplementary Tables S3–S6).

**Figure 2:**
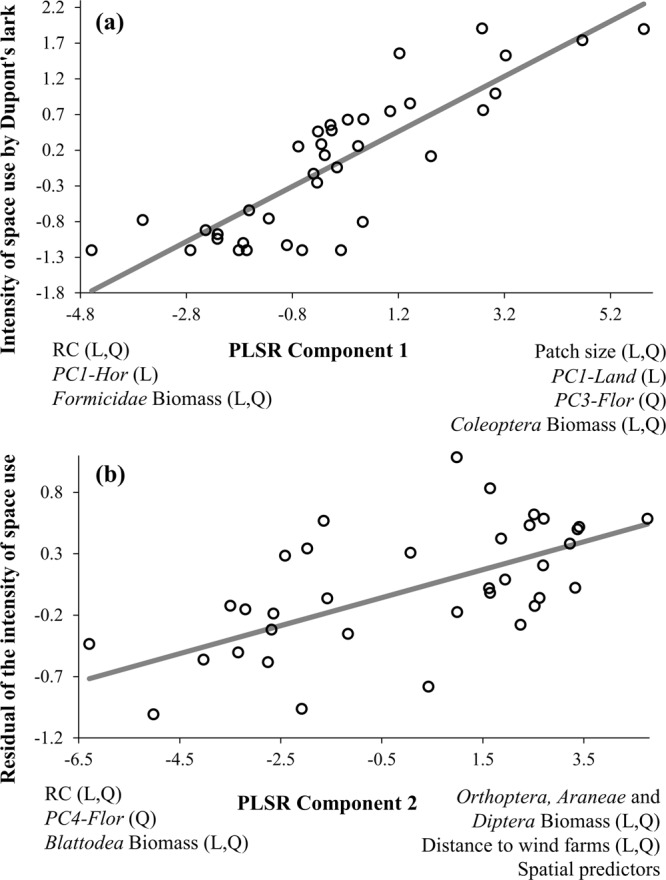
Results of the PLSR analysis incorporating 4 descriptors of habitat quality at metapopulation scale, 6 at landscape scale and 32 at microhabitat scale, and 9 spatial predictors: (**a**) relationship between the intensity of space use by Dupont’s lark in 2015 and the first component yielded by the PLSR; and (**b**) relationship between the residual variation in the intensity of space use after removing the effect of component 1 in (**a**), and the second PLSR component. The observations in 37 sampling stations (white dots) and model predictions (grey line) are depicted. In addition, linear (L) and/or quadratic (Q) relationships are specified. RC: relative connectivity index. PC1-*Hor*: first component yielded by the PCA on horizontal vegetation structure variables. PC3-*Flor* and PC4-*Flor*: third and fourth components yielded by the PCA on floristic composition variables. PC1-*Land*: first component yielded by the PCA on the land-use types.

**Figure 3:**
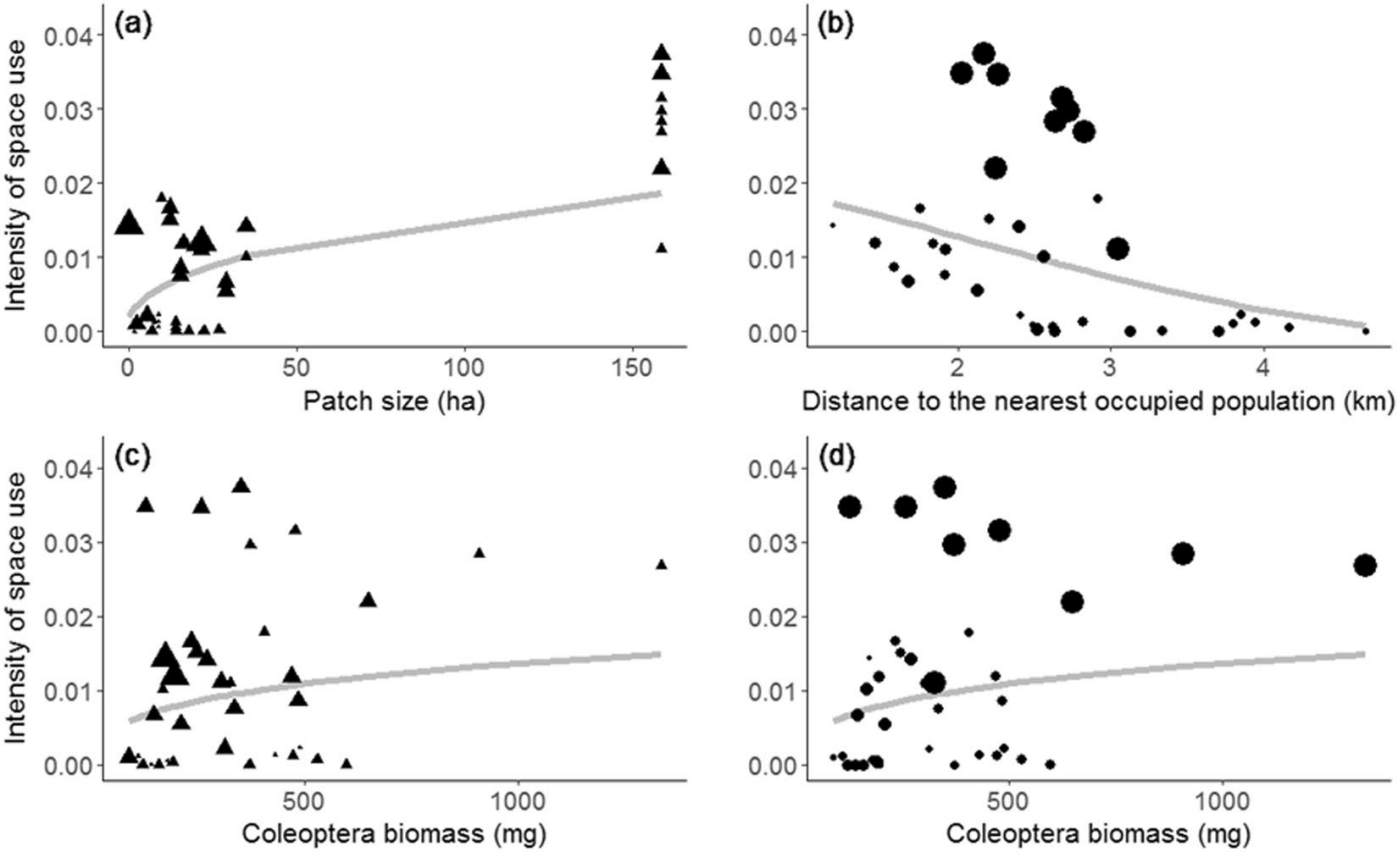
Relationships between the intensity of space use by Dupont’s lark and (**a**) patch size; (**b**) distance to the nearest occupied population (relative connectivity index RC); (**c**,**d**) *Coleoptera* biomass. Grey line depicts model predictions controlling by mean values for the other predictors incorporated in the PLSR. Differences in triangle size reflect differences in relative connectivity RC from isolated (smaller triangles) to connected (bigger) patches. Similarly, differences in circle size reflect differences in patch size, from small to large patches.

The PLSR analysing the effect of habitat quality at different spatial scales on the intensity of space use by Dupont’s lark yielded two components, which explained 71.19% (coefficient= 0.385, p <0.001) and 10.59% (coefficient= 0.139, p <0.001) of total variance in the response variable, respectively (total variance explained = 81.78%). The metapopulation variables had the highest contribution on the first PLSR component (sum of weights *ω*^2^ = 0.44), followed by the microhabitat (0.18) and the landscape variables (0.13) (Table 2). The first PLSR component was positively associated with patch size (linear and quadratic), *PC1-Land* (linear), *PC3-Flor* (quadratic) and *Coleoptera* biomass (linear and quadratic; Table 2; Fig. 2a). Conversely, it was negatively associated with the RC connectivity index (linear and quadratic), the *PC1-Hor* (linear) and the biomass of *Formicidae* (linear and quadratic; Table 2; Fig. 2a). Therefore, the intensity of space use by Dupont’s lark increased with the surface of optimal habitat (i.e. patch size and *PC1-Land*; Fig. 3a), the biomass of *Coleoptera* (Fig. 3c,d) and with a decreasing cover of the herbaceous genus *Stipa* (*PC3-Flor*). However, it decreased with the distance to the nearest occupied population (connectivity index RC; Fig. 3b), moss cover (*PC1-Hor*) and the biomass of *Formicidae* (Table 2; Fig. 2a).

**Table 2:** Results of the Partial Least Square Regressions (PLSR) analysing the relationship between descriptors of habitat quality at different spatial scales and the intensity of space use by Dupont’s lark in 37 sampling stations. Results are presented only for significant predictors with a square weight higher than 0.019 attending to *1/number of predictors* (see methods).

Predictors		PLSR Component 1		PLSR Component 2		*β*
		*ω*^2^	*ρ*	*ω*^2^	*ρ*	
Metapopulation	Relative connectivity index RC	**0.077**	**−0.387**	**0.041**	**−0.770**	−0.146
	Relative connectivity index RC^2^	**0.081**	**−0.436**	**0.030**	**−0.732**	−0.146
	Patch size	**0.122**	**0.781**	0.000	−0.108	0.156
	Patch size^2^	**0.156**	**0.820**	0.001	−0.090	0.179
Landscape	*PC1-Land* Land use types	**0.127**	**0.728**	0.002	−0.102	0.163
	Distance to Wind farms	0.002	−0.365	**0.061**	**0.680**	0.009
	Distance to Wind farms^2^	0.002	−0.373	**0.058**	**0.690**	0.005
Microhabitat	*PC1-Hor* Horizontal veg. structure	**0.036**	**−0.325**	0.007	0.136	−0.094
	*PC3-Flor* Floristic composition^2^	**0.046**	**0.453**	0.000	−0.068	0.097
	*PC4-Flor* Floristic composition^2^	0.002	0.282	**0.025**	**−0.160**	0.004
	*Coleoptera* biomass	**0.021**	**0.316**	0.000	−0.009	0.065
	*Coleoptera* biomass^2^	**0.021**	**0.316**	0.000	−0.014	0.063
	*Diptera* biomass	0.000	−0.239	**0.058**	**0.549**	0.033
	*Diptera* biomass^2^	0.006	−0.094	**0.054**	**0.472**	0.059
	*Hymenoptera - Formicidae* biomass	**0.029**	**−0.421**	0.002	−0.016	−0.070
	*Hymenoptera - Formicidae* biomass^2^	**0.025**	**−0.400**	0.002	0.000	−0.064
	*Araneae* biomass	0.007	−0.374	**0.030**	**0.386**	−0.017
	*Araneae* biomass^2^	0.004	−0.300	**0.020**	**0.267**	−0.013
	*Orthoptera* biomass	0.019	−0.536	**0.047**	**0.268**	−0.036
	*Orthoptera* biomass^2^	0.017	−0.502	**0.039**	**0.111**	−0.035
	*Blattodea* biomass	0.002	0.369	**0.060**	**−0.564**	−0.008
	*Blattodea* biomass^2^	0.002	0.384	**0.061**	**−0.585**	−0.006
Spatial	Y	0.007	−0.405	**0.041**	**0.762**	−0.014
	X	0.010	0.033	**0.028**	**0.685**	0.063
	XY	0.004	−0.379	**0.045**	**0.805**	−0.005
	X^2^	0.007	−0.406	**0.041**	**0.762**	−0.014
	Y^2^	0.010	0.033	**0.028**	**0.685**	0.063
	X^3^	0.007	−0.406	**0.041**	**0.762**	−0.014
	Y^3^	0.010	0.033	**0.028**	**0.686**	0.063
	X^2^Y	0.006	−0.393	**0.043**	**0.785**	−0.009
	XY^2^	0.003	−0.351	**0.048**	**0.835**	0.002

For each significant predictor, the correlation coefficient between the predictor and each PLSR component (*ρ*) and the square weights of each predictor (*ω*^2^), are shown. Moreover, the standardised regression coefficients (*β*) between the intensity of space use and each significant predictor, are given. See complete table with all predictors at metapopulation (4), landscape (6) and microhabitat scale (32), and spatial predictors (9) on Supplementary Table S7.

The second PLSR component works on the residual variation not explained by the first component (i.e., 100% of variance – 71.2% of variance explained by PLSR1 = 28.8%), accounting for 10.59% of total variance. The microhabitat variables had the highest contribution in the second PLSR component (sum of weights *ω*^2^ = 0.39), followed by the spatial predictors (0.34) and the landscape (0.12) and metapopulation variables (0.07). The second PLSR component was positively associated with the distance to wind farms (linear and quadratic), the biomass of *Diptera, Orthoptera* and *Araneae* (linear and quadratic) and all spatial predictors (Table 2; Fig. 2b). Conversely, it was negatively associated with the connectivity index RC (linear and quadratic), the *PC4-Flor* (quadratic) and the biomass of *Blattodea* (linear and quadratic; Table 2; Fig. 2b). Therefore, the intensity of space use by Dupont’s lark increased with the distance to wind farms and the biomass of *Diptera, Orthoptera* and *Araneae*. However, it decreased with the distance to the nearest occupied population (connectivity index RC), the cover of *Koeleria vallesiana* (*PC1-Hor*) and the biomass of *Blattodea* (Table 2; Fig. 2b).

The proportion of variation of Dupont’s lark intensity of space use accounted for each set of predictors, can be estimated using the weights and the proportion of variance explained by each component. Taking only the significant predictors in each PLSR component into account, the metapopulation variables explained 32% (PLSR1, 0.712 × 0.44 = 0.313; PLSR2, 0.106 × 0.07 = 0.007), microhabitat variables explained 16.9% (PLSR1, 0.712 × 0.18 = 0.128; PLSR2, 0.106 × 0.39 = 0.041) and landscape variables explained 10.5% of total variance in the response variable (PLSR1, 0.712 × 0.13 = 0.092; PLSR2, 0.106 × 0.12 = 0.013). Lastly, spatial predictors explained 3.6% of total variance (PLSR2, 0.106 × 0.34 = 0.036).

## Discussion

This research highlights the hierarchical nature of habitat-use by an endangered species, the Dupont’s lark. A previous study detected an effect of connectivity, patch size and landscape matrix on Dupont’s lark populations^8^. However, that work failed to detect an effect of habitat quality^8^ probably due to insufficient sample size at microhabitat scale to address variation in habitat patch quality. In our study, metapopulation factors had the greater explanatory power on the intensity of space use (32% of total explained variance), followed by microhabitat (16.9%), landscape (10.5%) and spatial predictors that control for spatial autocorrelation (3.6%). Thus, although factors at metapopulation and landscape scales are key drivers of habitat-use patterns, descriptors of habitat quality at microhabitat scale should not be overlooked. The multifactorial and multiscale nature of habitat-use has been described before in metapopulation dynamics (e.g. Jaquiéry *et al*.^7^), but multiscale approaches are seldom employed in research addressing habitat use patterns. A relevant finding of this work is the role of food availability in habitat use patterns by Dupont’s lark, along with classic metapopulation factors (i.e. connectivity and patch size). In addition, the multiscale approach employed might be applicable in studies addressing distribution patterns or habitat selection processes in other species.

Connectivity and patch size are two key factors affecting metapopulation dynamics and distribution patterns, being more evident as the dispersal capacity of the study species decreases^4^. In particular, our study species has been described as a strongly territorial bird with short-distance movements (between 1 and 5 km^23,40–42^) and short-medium dispersal distances in juveniles (30–40 km^43^). In this study, the intensity of space use by Dupont’s lark increased with patch size and decreased with isolation. These results are consistent with previously described processes under the conceptual framework of metapopulation theory: (1) higher recolonization probability in larger and connected patches^4^; and (2) higher vulnerability to stochastic processes (demographic, environmental or genetic drift) in smaller and isolated populations^44,45^; and, in particular, with previous studies on Dupont’s lark populations^8,14^. Fixing the other predictors at a mean value, our model predicts a decelerated increase in the intensity of space use in patches up to 50 ha, from which the trend becomes smoother (Fig. 3a). This patch size threshold might be suggesting a ‘crowding effect’ in smaller and isolated patches as it has been previously pointed out^8^. Nevertheless, it should not be taken as a minimum patch size threshold, since the intensity of space use continues to increase up to 150 ha (Fig. 3a) and we ignore the effect above this size. Moreover, the absence of patches between 35 and 150 ha may be driving the accelerated increase of the intensity of space use above 50 ha. On the other hand, we were not able to identify a threshold distance to the nearest occupied population, since our results suggest that this effect has a monotonic response on the intensity of space use (Fig. 3b). This could be an effect related to landscape configuration, since the level of connectivity among patches in the study area is high (distances ranging from 1 to 5 km to the nearest occupied population; Fig. 3b) in accordance with distance thresholds described before (30 km for genetic differentiation)^45^ and with juvenile dispersal events recorded up to 33 km^43^. However, the intensity of space use by Dupont’s lark decreased drastically in small patches located more than 3 km away from the nearest population (Fig. 3b). This suggests a joint effect of patch size and connectivity that might be enhanced by the limited dispersal propensity of the study species.

Human disturbances also affect species distribution patterns^46^. It is well-known that intensive agricultural practices^16^ as well as wind farms (e.g. Northrup & Wittemyer^47^) have deleterious effects on bird populations. In this study, the absence of variability in the proximity to crops may have prevented the detection of direct effects (Mean ± SD = 163.33 ± 161.79 m). In addition, the effects of agricultural practices (e.g. use of agrochemicals) might propagate at larger spatial scales than the immediate surroundings and over long periods of time^48^. Thus future studies should compare shrub-steppe patches under different agricultural contexts. On the other hand, the intensity of space use by the Dupont’s lark increased with the distance to wind farms, in accordance with a previous study which identified a 4.5 km distance threshold to wind farms guaranteeing the persistence of Dupont’s lark populations^14^. The weak explanatory power of this predictor (percentage of variation explained = 1.13%) might be due to the sampling design of this research, since sampling stations were located in the absence of wind farms and thus, close and far distances to wind farms were not sufficiently covered (Range = 0.85–4.61 km). In any case, these results support previous findings on negative impacts of wind farms on Dupont’s lark populations up to 4.5 km^14^.

At microhabitat scale, food availability is a key factor influencing population dynamics and distribution patterns of species (e.g. Perrig *et al*.^49^ or Zengeya *et al*.^50^). Specifically, it affects survival and reproductive success in insectivorous bird species, with a direct impact on population density^51,52^. In accordance with the first PLSR component, the intensity of space use by Dupont’s lark decreased with *Formicidae* biomass and increased with *Coleoptera* biomass, which seems to be relevant in smaller and isolated patches (Fig. 3c,d). Beetles are a crucial group in agroecosystems due to their predominance (30.24% of total biomass in this study) and high nutritive value^53^, and their role on habitat selection processes has been previously described for other steppe-birds^29,54^. Particularly, the order *Coleoptera* has been described as important on the diet of Dupont’s lark nestlings^19^. Information about adults’ diet is lacking, but the relationship between beetles and Dupont’s lark has been previously established^16,20,55^. On the other hand, the negative effect of ants might not result from a direct rejection by Dupont’s lark. It could rather be explained by a relationship mediated by other taxa relevant in its diet. For instance, the high territoriality and aggressiveness of ants have important effects on other epigean arthropods^56,57^ and therefore, Dupont’s lark might be selecting the most nutritive ones (e.g. *Coleoptera*), which would be avoiding territorial ants. In any case, more information is needed in this aspect. Lastly, other arthropod groups had an effect on the intensity of space use by the Dupont’s lark, but in a lower magnitude (i.e., second PLSR component). The biomass of *Orthoptera*, *Diptera* and *Araneae* was positively associated with the intensity of space use, which is in agreement with previous studies highlighting their role on the diet of steppe-birds^54^ and specifically on the diet of Dupont’s lark nestlings^19^. However, these results should be taken with caution since the incongruences between the correlation and regression coefficients (Table 2) reveal high instability on the global effect of some of these predictors on the intensity of space use by Dupont’s lark (e.g. *Orthoptera* and *Araneae*; Table 2). Lastly, the negative effect of *Blattodea* biomass (1.2% of variance explained) on space use by the Dupont’s lark is hard to interpret and could be mediated by the effect of other taxa or factors not addressed in this study.

The abandonment of livestock grazing leads to the loss of shrub-steppe associations^16^ and might reduce the availability of coprophagous arthropods, potential food source for insectivorous bird species^20^. However, the abandonment of extensive livestock is a generalized phenomenon in the study area^55^ reducing the variability in both predictors (coprophagous biomass and herbivore abundance), and hindering the detection of effects on the intensity of space use (Mean ± SD= 226.3 ± 168.7 mg and 1.9 ± 2.8 herbivore droppings, respectively). Therefore, future research should focus on addressing these patterns at a greater scale and comparing areas with different livestock densities.

Lastly, vegetation structure and floristic composition are two additional factors widely employed in the description of habitat quality^58^. In this work, sampling stations were located in areas fulfilling the requirements of this habitat specialist (i.e. areas with medium-sized shrubs)^11^. This fact explains the null effect of the majority of these variables on the intensity of space use, except for the negative effect of moss cover (*PC1-Hor*) and the herbaceous taxa *Stipa spp*. (PC3-*Flor*) and *Koeleria vallesiana* cover (PC4-*Flor*). Moss and herbaceous cover makes pedestrian habits and food search more difficult, which could explain its negative effect on the intensity of space use by Dupont’s lark.

One potential pitfall of our study design is the absence of patches between 35 and 150 ha, and the low number of patches sampled above 150 ha (one single patch with 9 sampling stations), which may be driving the observed results (Fig. 3a,b). Future studies should cover the whole patch gradient and increase sample size in order to be more representative. In any case, the analytical approach employed allowed us to control for spatial dependency and obtain conclusive results, highlighting the multifactorial and hierarchical nature of habitat-use by species inhabiting fragmented landscapes, such as Dupont’s lark. Large and connected patches are crucial in the conservation of Dupont’s lark since they impact on population density, extinction risk, occurrence^8,14^, and therefore habitat-use patterns. However, other descriptors of habitat quality at microhabitat scale should not be neglected. In this study large and connected patches were not necessarily the ones with better quality habitats in terms of food availability (*Coleoptera* biomass; Fig. 3c,d), suggesting that multiscale factors contribute jointly to explain the distribution patterns of Dupont’s lark. Therefore, conservation measures should take into account the effect of habitat quality at all spatial scales: ensuring the connectivity, guaranteeing a minimum patch size, and improving habitat quality within patches (food availability and vegetation structure).

These results have important implications for the conservation of Dupont’s lark, and may also benefit other bird species linked to shrub-steppe habitats. First, conservation measures aimed to enhance the connectivity and increase habitat availability must be prioritized since they explained 32% of the variance on space use. Tree plantations and failed afforestation subsidized by the Common Agricultural Policy (CAP) of the European Union are common causes of habitat loss in Spanish shrub-steppes^10,16^. Therefore, habitat restoration through tree removal is a potential conservation measure to reduce isolation and increase habitat availability (see for example conservation measures carried out by LIFE-Ricoti project www.lifericoti.org). Secondly, specific actions can be designed to increase habitat patch quality. Extensive grazing plays a key role in the maintenance of Spanish shrub-steppes and its avifauna, preserving the plant structure^17^ and potentially increasing food availability^18^. Clearing shrubs or the establishment of grazing regimes through voluntary agreements under territorial custody programs, are recommended actions to simulate extensive grazing and preserve habitat quality in shrub-steppes. However, the improvement of policies and aids of the CAP committed to maintain the traditional practice of extensive livestock must be prioritized, incorporating grazed shrublands as eligible land for direct payments.

## Supplementary information

Supplementary Information

## Data Availability

Data used in the current study is available at: 10.6084/m9.figshare.11301752.v1.
