# Supplementary material for: Hierarchical habitat-use by an endangered steppe bird in fragmented landscapes is associated with large connected patches and high food availability
Source: Sci Rep. 2019 Dec 12;9:19010. doi: 10.1038/s41598-019-55467-2 (PMC6908678; doi:10.1038/s41598-019-55467-2)
Supplement: Supplementary file 1 — Supplementary Information [file 41598_2019_55467_MOESM1_ESM.docx]

**Hierarchical habitat-use by an endangered steppe bird in fragmented landscapes is associated with large connected patches and high food availability**

*Scientific Reports*

Julia Gómez-Catasús ^a,b*^, Vicente Garza ^a,c^, Manuel B. Morales ^a,b^ and Juan Traba ^a,b^

^a^ Terrestrial Ecology Group, Department of Ecology, Universidad Autónoma de Madrid (TEG-UAM). C/ Darwin 2, 28049 Madrid, Spain

^b^ Centro de Investigación en Biodiversidad y Cambio Global, Universidad Autónoma de Madrid (CIBC-UAM). C/ Darwin 2, 28049 Madrid, Spain

^c^ C/ Vía Límite 29, 28029 Madrid, Spain

^*^ Corresponding author: Julia Gómez-Catasús. C/ Darwin 2, 28049 Madrid, Spain. E-mail: [julia.gomez@uam.es](mailto:julia.gomez@uam.es); Telephone number: +34 639 58 24 95. ORCID: 0000-0001-8949-5318.


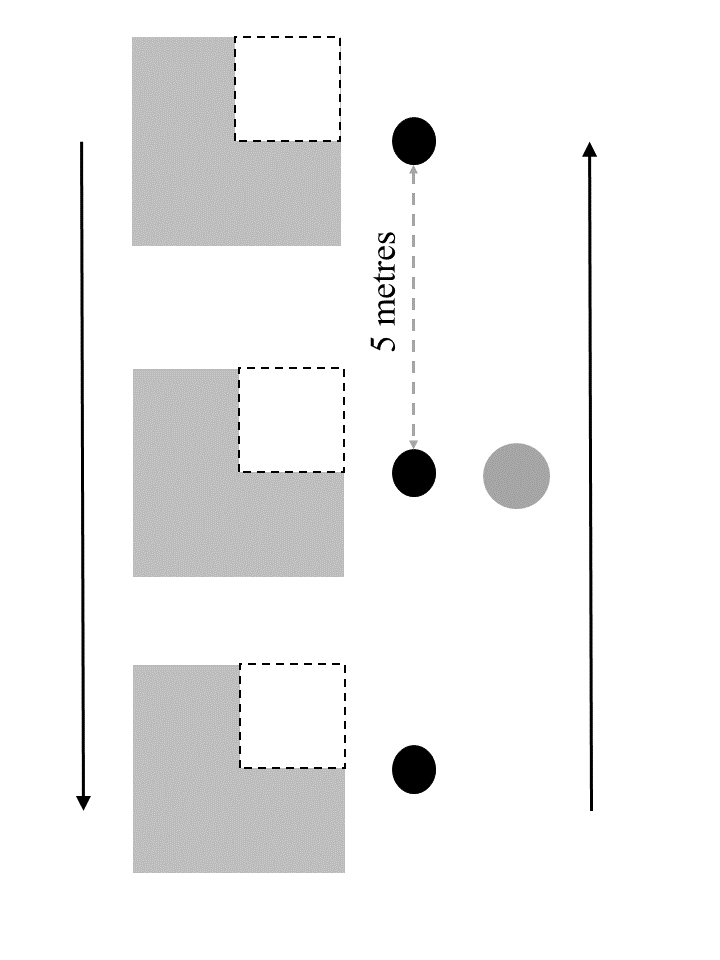


**Figure S1.** Scheme showing the structure of each sampling station. Each sampling station consisted of: (1) three pitfall traps (black dots); (2) one coprophagous arthropods trap (grey dot); (3) three quadrats of 2 x 2 m (grey squares) where the herbivore abundance sampling was carried out; (4) three quadrats of 1 x 1 m (white striped squares) for the vegetation sampling; and (5) two transects of 10 m length where flying arthropods were sampled (black arrows).

**Table S1.** Results for the correlation analysis between variables incorporated as predictors in the PLSR. The Pearson correlation coefficients (PC) and the p-value (P) are shown. Significant PCs with a p-value lower than 0.01 were marked with two asterisks (**), whereas those significant PCs under a 0.05 significance threshold were marked with one asterisk (*). Each predictor is named as: 1) Relative connectivity index RC; 2) Patch size; 3) Land-use variables (*PC1-Land*); 4) Distance to wind farms; 5) Distance to wind crops; 6) Herbivore abundance; 7) Horizontal plant structure – First component (*PC1-Hor*); 8) Horizontal plant structure – Second component (*PC2-Hor*); 9) Vertical plant structure – First component (*PC1-Ver*); 10) Vertical plant structure – Second component (*PC2-Ver*); 11) Floristic composition – First component (*PC1-Flor*); 12) Floristic composition – Second component (*PC2-Flor*); 13) Floristic composition – Third component (*PC3-Flor*); 14) Floristic composition – Fourth component (*PC4-Flor*); 15) Biomass of coprophagous arthropods; 16) Biomass of *Coleoptera*; 17) Biomass of *Diptera*; 18) Biomass of *Formicidae*; 19) Biomass of *Araneae*; 20) Biomass of *Orthoptera*; 21) Biomass of *Blattodea*.

| **Variables** | | **1** | **2** | **3** | **4** | **5** | **6** | **7** | **8** |
| --- | --- | --- | --- | --- | --- | --- | --- | --- | --- |
| 1 | PC | **1** |  |  |  |  |  |  |  |
|  | P |  |  |  |  |  |  |  |  |
| 2 | PC | -.146 | **1** |  |  |  |  |  |  |
|  | P | .389 |  |  |  |  |  |  |  |
| 3 | PC | -.087 | ,648^**^ | **1** |  |  |  |  |  |
|  | P | .611 | .000 |  |  |  |  |  |  |
| 4 | PC | -,350^*^ | -,432^**^ | -,381^*^ | **1** |  |  |  |  |
|  | P | .033 | .008 | .020 |  |  |  |  |  |
| 5 | PC | -.141 | -.148 | .007 | -.206 | **1** |  |  |  |
|  | P | .404 | .382 | .969 | .222 |  |  |  |  |
| 6 | PC | .119 | .268 | .054 | -.056 | -.258 | **1** |  |  |
|  | P | .482 | .109 | .751 | .741 | .123 |  |  |  |
| 7 | PC | -.037 | -.153 | -,392^*^ | ,360^*^ | -,347^*^ | .227 | **1** |  |
|  | P | .828 | .367 | .017 | .029 | .035 | .176 |  |  |
| 8 | PC | .105 | .213 | .131 | -.083 | -.074 | .270 | .000 | **1** |
|  | P | .537 | .206 | .438 | .626 | .664 | .107 | 1.000 |  |
| 9 | PC | -,407^*^ | -.160 | .098 | .200 | -.074 | -.273 | -.017 | -.311 |
|  | P | .012 | .343 | .564 | .236 | .665 | .102 | .919 | .061 |
| 10 | PC | .066 | .158 | .157 | -.042 | -.021 | .037 | .187 | -.203 |
|  | P | .700 | .350 | .353 | .807 | .903 | .829 | .267 | .229 |
| 11 | PC | .099 | .142 | .192 | -.232 | .301 | -.272 | -,499^**^ | -.154 |
|  | P | .558 | .403 | .254 | .167 | .070 | .104 | .002 | .362 |
| 12 | PC | -,337^*^ | -.226 | -.208 | ,571^**^ | -.138 | -.157 | ,325^*^ | .094 |
|  | P | .041 | .179 | .217 | .000 | .416 | .353 | .050 | .581 |
| 13 | PC | .133 | -,326^*^ | -.245 | .175 | -.151 | -.192 | -.021 | -.222 |
|  | P | .432 | .049 | .143 | .301 | .372 | .255 | .904 | .187 |
| 14 | PC | .218 | .035 | -.272 | .013 | -.187 | .139 | .104 | -.029 |
|  | P | .195 | .838 | .104 | .941 | .267 | .413 | .541 | .866 |
| 15 | PC | -.180 | -.001 | .047 | .072 | .199 | .002 | -.100 | -.018 |
|  | P | .285 | .994 | .781 | .670 | .239 | .993 | .554 | .915 |
| 16 | PC | .001 | ,359^*^ | .116 | -.006 | -.211 | -.140 | .001 | .039 |
|  | P | .995 | .029 | .496 | .972 | .211 | .409 | .997 | .818 |
| 17 | PC | -.195 | -.290 | -.267 | ,345^*^ | .058 | -.122 | .066 | .181 |
|  | P | .248 | .081 | .111 | .036 | .735 | .473 | .697 | .284 |
| 18 | PC | .237 | -.147 | -.204 | .016 | .033 | -.018 | -.065 | .218 |
|  | P | .157 | .386 | .227 | .924 | .844 | .914 | .701 | .194 |
| 19 | PC | -.079 | -.139 | -.150 | .178 | -.073 | .075 | .131 | .030 |
|  | P | .642 | .412 | .374 | .292 | .669 | .661 | .439 | .859 |
| 20 | PC | .054 | -.267 | -.260 | .153 | -.101 | .037 | .101 | .205 |
|  | P | .753 | .110 | .121 | .367 | .550 | .830 | .553 | .224 |
| 21 | PC | .159 | .273 | .291 | -,418^**^ | -.060 | -.015 | -.196 | -.247 |
|  | P | .348 | .101 | .081 | .010 | .726 | .931 | .245 | .140 |

**Table S2.** [Table S1 continuation]. Results for the correlation analysis between variables incorporated as predictors in the PLSR. The Pearson correlation coefficients (PC) and the p-value (P) are shown. Significant PCs with a p-value lower than 0.01 were marked with two asterisks (**), whereas those significant PCs under a 0.05 significance threshold were marked with one asterisk (*). Each predictor is named as: 1) Relative connectivity index RC; 2) Patch size; 3) Land-use variables (*PC1-Land*); 4) Distance to wind farms; 5) Distance to wind crops; 6) Herbivore abundance; 7) Horizontal plant structure – First component (*PC1-Hor*); 8) Horizontal plant structure – Second component (*PC2-Hor*); 9) Vertical plant structure – First component (*PC1-Ver*); 10) Vertical plant structure – Second component (*PC2-Ver*); 11) Floristic composition – First component (*PC1-Flor*); 12) Floristic composition – Second component (*PC2-Flor*); 13) Floristic composition – Third component (*PC3-Flor*); 14) Floristic composition – Fourth component (*PC4-Flor*); 15) Biomass of coprophagous arthropods; 16) Biomass of *Coleoptera*; 17) Biomass of *Diptera*; 18) Biomass of *Formicidae*; 19) Biomass of *Araneae*; 20) Biomass of *Orthoptera*; 21) Biomass of *Blattodea*.

| **Variables** | | **9** | **10** | **11** | **12** | **13** | **14** | **15** | **16** | **17** | **18** | **19** | **20** |
| --- | --- | --- | --- | --- | --- | --- | --- | --- | --- | --- | --- | --- | --- |
| 9 | PC | **1** |  |  |  |  |  |  |  |  |  |  |  |
|  | P |  |  |  |  |  |  |  |  |  |  |  |  |
| 10 | PC | .000 | **1** |  |  |  |  |  |  |  |  |  |  |
|  | P | 1.00 |  |  |  |  |  |  |  |  |  |  |  |
| 11 | PC | .089 | -.157 | **1** |  |  |  |  |  |  |  |  |  |
|  | P | .600 | .352 |  |  |  |  |  |  |  |  |  |  |
| 12 | PC | ,356^*^ | -.166 | .000 | **1** |  |  |  |  |  |  |  |  |
|  | P | .031 | .327 | 1.000 |  |  |  |  |  |  |  |  |  |
| 13 | PC | -.108 | -.183 | .000 | .000 | **1** |  |  |  |  |  |  |  |
|  | P | .523 | .278 | 1.000 | 1.000 |  |  |  |  |  |  |  |  |
| 14 | PC | -.266 | -.203 | .000 | .000 | .000 | **1** |  |  |  |  |  |  |
|  | P | .111 | .228 | 1.000 | 1.000 | 1.000 |  |  |  |  |  |  |  |
| 15 | PC | .045 | -.220 | -.001 | .002 | -.177 | -.153 | **1** |  |  |  |  |  |
|  | P | .791 | .190 | .994 | .989 | .296 | .367 |  |  |  |  |  |  |
| 16 | PC | -.208 | .101 | .205 | .113 | .188 | .069 | -.155 | **1** |  |  |  |  |
|  | P | .216 | .550 | .224 | .507 | .266 | .686 | .359 |  |  |  |  |  |
| 17 | PC | .149 | -,339^*^ | .068 | ,445^**^ | .026 | .152 | -.221 | -.028 | **1** |  |  |  |
|  | P | .379 | .040 | .688 | .006 | .879 | .370 | .190 | .870 |  |  |  |  |
| 18 | PC | .015 | -.148 | .000 | .095 | -,440^**^ | .138 | .043 | -.130 | .105 | **1** |  |  |
|  | P | .928 | .381 | .999 | .575 | .006 | .416 | .799 | .443 | .535 |  |  |  |
| 19 | PC | .085 | -.202 | -.084 | .147 | .034 | .095 | .096 | -.087 | .302 | .162 | **1** |  |
|  | P | .615 | .229 | .622 | .385 | .842 | .574 | .571 | .608 | .069 | .338 |  |  |
| 20 | PC | -.089 | .037 | -.284 | -.005 | .084 | .086 | -.139 | -.059 | .218 | .293 | ,583^**^ | **1** |
|  | P | .602 | .828 | .088 | .976 | .620 | .612 | .413 | .727 | .195 | .079 | .000 |  |
| 21 | PC | .010 | .298 | .038 | -.284 | .144 | -.194 | .090 | .046 | -,670^**^ | -.297 | -,527^**^ | -,488^**^ |
|  | P | .951 | .074 | .822 | .088 | .394 | .250 | .596 | .786 | .000 | .075 | .001 | .002 |

**Table S3.** Results of the Principal Component Analysis (PCA) on land-use variables. The correlation coefficients between each variable and the PCA component are shown. Significant correlation coefficients (p<0.001) are marked with asterisk (*).

| Variable | PC1-Land |
| --- | --- |
| Crops (%) | -0.201 |
| Infrastructures (%) | -0.130 |
| Shrub-steppe with slope < 15% (%) | 0.978* |
| Shrub-steppe with slope > 15% (%) | -0.807* |
| Pasture (%) | -0.489 |
| Afforestation (%) | 0.197 |
| Ploughing | -0.239 |
| Variance explained | 58.67 % |

**Table S4.** Results of the Principal Component Analysis (PCA) on horizontal plant structure variables. The correlation coefficients between each variable and the PCA components are shown. Significant correlation coefficients (p<0.001) are marked with asterisk (*).

| Variable | PC1-Hor | PC2-Hor |
| --- | --- | --- |
| Rock cover (%) | -0.533 | 0.776* |
| Moss cover (%) | 0.920* | 0.324 |
| Total vegetation cover (%) | 0.270 | 0.459 |
| Bare ground cover (%) | 0.398 | 0.266 |
| Shrub cover (%) | 0.320 | 0.202 |
| Perennial herbaceous cover (%) | 0.007 | -0.307 |
| Annual herbaceous cover (%) | 0.150 | 0.559 |
| Detritus cover (%) | 0.504 | -0.046 |
| Variance explained | 35.63 % | 24.74 % |
| Total variance explained | 35.63 % | 60.37 % |

**Table S5.** Results of the Principal Component Analysis (PCA) on vertical plant structure variables. The correlation coefficients between each variable and the PCA component are shown. Significant correlation coefficients (p<0.001) are marked with asterisk (*).

| Variable | PC1-Ver | PC2-Ver |
| --- | --- | --- |
| Number of contacts at 0-5 cm height | 0.540 | -0.629 |
| Number of contacts at 5-10 cm height | 0.843* | -0.327 |
| Number of contacts at 10-30 cm height | 0.864* | 0.019 |
| Number of contacts above 30 cm height | 0.423 | 0.783* |
| Maximum modal height (cm) | 0.494 | 0.543 |
| Variance explained | 43.45 % | 28.23 % |
| Total variance explained | 43.45 % | 71.69 % |

**Table S6.** Results of the Principal Component Analysis (PCA) on floristic composition variables. The correlation coefficients between each variable and the PCA component are shown. Significant correlation coefficients (p<0.001) are marked with asterisk (*).

| Variable* | PC1-Flor | PC2-Flor | PC3-Flor | PC4-Flor |
| --- | --- | --- | --- | --- |
| *Anthyllis vulneraria* | 0.040 | -0.049 | -0.108 | -0.052 |
| *Aphyllanthes monspeliensis* | 0.127 | -0.501 | 0.195 | 0.011 |
| *Artemisia assoana* | 0.015 | 0.073 | -0.258 | -0.010 |
| *Eryngium campestre* | -0.222 | 0.001 | -0.134 | 0.119 |
| *Euphorbia paralias* | -0.271 | 0.133 | 0.184 | 0.092 |
| *Festuca sp* | -0.273 | -0.424 | 0.164 | 0.270 |
| *Festuca ovina* | 0.496 | 0.346 | -0.036 | 0.214 |
| *Festuca rubra* | 0.082 | 0.283 | 0.123 | -0.003 |
| *Fumana ericoides* | 0.106 | -0.181 | 0.010 | -0.460 |
| *Fumana procumbens* | 0.187 | 0.209 | 0.012 | 0.179 |
| *Genista pumila* | 0.107 | -0.269 | -0.532 | -0.494 |
| *Genista scorpius* | -0.317 | 0.494 | 0.420 | -0.363 |
| *Graminea unid* | 0.055 | 0.062 | 0.142 | 0.282 |
| *Helianthemum sp* | -0.284 | -0.503 | -0.020 | -0.217 |
| *Helianthemum apenninum* | 0.390 | 0.269 | -0.063 | 0.308 |
| *Helianthemum cinereum* | 0.466 | 0.296 | -0.001 | 0.272 |
| *Koeleria vallesiana* | 0.232 | 0.246 | -0.258 | 0.695* |
| *Lavandula latifolia* | 0.208 | -0.474 | 0.463 | -0.028 |
| *Lithodora fruticosa* | 0.539 | 0.086 | 0.108 | -0.487 |
| *Marrubium supinum* | -0.118 | 0.122 | -0.015 | 0.280 |
| *Marrubium vulgare* | -0.165 | 0.070 | -0.144 | 0.217 |
| *Medicago sativa* | -0.313 | -0.026 | -0.051 | -0.069 |
| *Phlomis lychnitis* | -0.154 | -0.028 | -0.173 | 0.454 |
| *Satureja montana* | 0.223 | -0.236 | 0.345 | 0.116 |
| *Sedum brevifolium* | 0.156 | 0.105 | 0.006 | 0.176 |
| *Sedum sediforme* | 0.207 | 0.143 | -0.195 | -0.115 |
| *Staehelina dubia* | -0.111 | 0.067 | 0.265 | -0.301 |
| *Stipa sp* | -0.216 | -0.120 | -0.843* | -0.060 |
| *Stipa barbata* | -0.205 | 0.173 | 0.217 | -0.207 |
| *Teucrium sp.* | 0.320 | 0.215 | -0.091 | 0.122 |
| *Teucrium chamaedrys* | 0.023 | -0.172 | -0.080 | -0.050 |
| *Teucrium polium* | 0.149 | 0.198 | -0.059 | -0.154 |
| *Thymus mastigophorus* | 0.121 | -0.817* | 0.374 | 0.152 |
| *Thymus vulgaris* | 0.833* | 0.332 | 0.143 | -0.041 |
| *Thymus zygis* | -0.890* | 0.301 | 0.237 | -0.014 |
| Variance explained | 22.79 % | 13.29 % | 11.87 % | 8.27 % |
| Total variance explained | 22.79 % | 36.08 % | 47.96 % | 56.23 % |
| * All variables were measured in percentage of specific cover (%) | | | | |

**Table S7.** Results of the Partial Least Square Regressions (PLSR) analysing the relationship between descriptors of habitat quality at different spatial scales and the intensity of space use by Dupont’s lark in 37 sampling stations. For each array of variables at metapopulation (4), landscape (6) and microhabitat scale (32), and spatial predictors (9), the correlation coefficient between the predictor and each PLSR component ($\rho$) and the square weights of each predictor ($\omega^{2}$), are shown. Moreover, the standardised regression coefficients ($\beta$) between the intensity of space use and each significant predictor, are given. Significant predictors with a square weight higher than 0.019 attending to *1/number of predictors* (see methods), are marked in bold.

|  | **Predictors** | **PLSR Component 1** | | **PLSR Component 2** | | | $\boldsymbol{\beta}$ |
| --- | --- | --- | --- | --- | --- | --- | --- |
|  |  | $\boldsymbol{\omega}^{\boldsymbol{2}}$ | $\boldsymbol{\rho}$ | | $\boldsymbol{\omega}^{\boldsymbol{2}}$ | $\boldsymbol{\rho}$ |  |
| Metapopulation | **Relative connectivity index RC** | **0.077** | **-0.387** | | **0.041** | **-0.770** | **-**0.146 |
|  | **Relative connectivity index RC^2^** | **0.081** | **-0.436** | | **0.030** | **-0.732** | -0.146 |
|  | **Patch size** | **0.122** | **0.781** | | 0.000 | -0.108 | 0.156 |
|  | **Patch size^2^** | **0.156** | **0.820** | | 0.001 | -0.090 | 0.179 |
| Landscape | ***PC1-Land* Land use types** | **0.127** | **0.728** | | 0.002 | -0.102 | 0.163 |
|  | *PC1-Land* Land use types^2^ | 0.002 | 0.108 | | 0.000 | 0.142 |  |
|  | **Distance to Wind farms** | 0.002 | -0.365 | | **0.061** | **0.680** | 0.009 |
|  | **Distance to Wind farms^2^** | 0.002 | -0.373 | | **0.058** | **0.690** | 0.005 |
|  | Distance to Crops | 0.006 | 0.161 | | 0.000 | -0.012 |  |
|  | Distance to Crops^2^ | 0.005 | -0.150 | | 0.000 | 0.060 |  |
| Microhabitat | Herbivore abundance | 0.000 | 0.001 | | 0.001 | -0.157 |  |
|  | Herbivore abundance^2^ | 0.000 | -0.079 | | 0.003 | -0.105 |  |
|  | ***PC1-Hor* Horizontal veg. structure** | **0.036** | **-0.325** | | 0.007 | 0.136 | -0.094 |
|  | *PC1-Hor* Horizontal veg. structure^2^ | 0.009 | 0.074 | | 0.016 | 0.380 |  |
|  | *PC2-Hor* Horizontal veg. structure | 0.000 | 0.026 | | 0.000 | 0.057 |  |
|  | *PC2-Hor* Horizontal veg. structure^2^ | 0.004 | -0.138 | | 0.000 | -0.186 |  |
|  | *PC1-Ver* Vertical veg. structure | 0.003 | 0.033 | | 0.006 | 0.438 |  |
|  | *PC1-Ver* Vertical veg. structure^2^ | 0.001 | -0.111 | | 0.001 | 0.058 |  |
|  | *PC2-Ver* Vertical veg. structure | 0.003 | 0.196 | | 0.004 | -0.135 |  |
|  | *PC2-Ver* Vertical veg. structure^2^ | 0.009 | 0.176 | | 0.001 | 0.232 |  |
|  | *PC1-Flor* Floristic composition | 0.013 | 0.260 | | 0.000 | -0.092 |  |
|  | *PC1-Flor* Floristic composition^2^ | 0.015 | -0.334 | | 0.004 | 0.372 |  |
|  | *PC2-Flor* Floristic composition | 0.000 | -0.149 | | 0.010 | 0.538 |  |
|  | *PC2-Flor* Floristic composition^2^ | 0.002 | 0.155 | | 0.002 | -0.288 |  |
|  | *PC3-Flor* Floristic composition | 0.017 | -0.282 | | 0.000 | -0.090 |  |
|  | ***PC3-Flor* Floristic composition^2^** | **0.046** | **0.453** | | 0.000 | -0.068 | 0.097 |
|  | *PC4-Flor* Floristic composition | 0.021 | -0.315 | | 0.000 | -0.076 |  |
|  | ***PC4-Flor* Floristic composition^2^** | 0.002 | 0.282 | | **0.025** | **-0.160** | 0.004 |
|  | Dung arthropods biomass | 0.009 | 0.113 | | 0.007 | 0.160 |  |
|  | Dung arthropods biomass^2^ | 0.008 | 0.116 | | 0.005 | 0.111 |  |
|  | ***Coleoptera* biomass** | **0.021** | **0.316** | | 0.000 | -0.009 | 0.065 |
|  | ***Coleoptera* biomass^2^** | **0.021** | **0.316** | | 0.000 | -0.014 | 0.063 |
|  | ***Diptera* biomass** | 0.000 | -0.239 | | **0.058** | **0.549** | 0.033 |
|  | ***Diptera* biomass^2^** | 0.006 | -0.094 | | **0.054** | **0.472** | 0.059 |
|  | ***Hymenoptera - Formicidae* biomass** | **0.029** | **-0.421** | | 0.002 | -0.016 | -0.070 |
|  | ***Hymenoptera - Formicidae* biomass^2^** | **0.025** | **-0.400** | | 0.002 | 0.000 | -0.064 |
|  | ***Araneae* biomass** | 0.007 | -0.374 | | **0.030** | **0.386** | -0.017 |
|  | ***Araneae* biomass^2^** | 0.004 | -0.300 | | **0.020** | **0.267** | -0.013 |
|  | ***Orthoptera* biomass** | 0.019 | -0.536 | | **0.047** | **0.268** | -0.036 |
|  | ***Orthoptera* biomass^2^** | 0.017 | -0.502 | | **0.039** | **0.111** | -0.035 |
|  | ***Blattodea* biomass** | 0.002 | 0.369 | | **0.060** | **-0.564** | -0.008 |
|  | ***Blattodea* biomass^2^** | 0.002 | 0.384 | | **0.061** | **-0.585** | -0.006 |
| Spatial | **Y** | 0.007 | -0.405 | | **0.041** | **0.762** | -0.014 |
|  | **X** | 0.010 | 0.033 | | **0.028** | **0.685** | 0.063 |
|  | **XY** | 0.004 | -0.379 | | **0.045** | **0.805** | -0.005 |
|  | **X^2^** | 0.007 | -0.406 | | **0.041** | **0.762** | -0.014 |
|  | **Y^2^** | 0.010 | 0.033 | | **0.028** | **0.685** | 0.063 |
|  | **X^3^** | 0.007 | -0.406 | | **0.041** | **0.762** | -0.014 |
|  | **Y^3^** | 0.010 | 0.033 | | **0.028** | **0.686** | 0.063 |
|  | **X^2^Y** | 0.006 | -0.393 | | **0.043** | **0.785** | -0.009 |
|  | **XY^2^** | 0.003 | -0.351 | | **0.048** | **0.835** | 0.002 |
